# MucR from *Sinorhizobium meliloti*: New Insights into Its DNA Targets and Its Ability to Oligomerize

**DOI:** 10.3390/ijms241914702

**Published:** 2023-09-29

**Authors:** Martina Slapakova, Domenico Sgambati, Luciano Pirone, Veronica Russo, Gianluca D’Abrosca, Mariangela Valletta, Rosita Russo, Angela Chambery, Gaetano Malgieri, Emilia Maria Pedone, Remus Thei Dame, Paolo Vincenzo Pedone, Ilaria Baglivo

**Affiliations:** 1 Department of Environmental, Biological and Pharmaceutical Sciences and Technologies, University of Campania “Luigi Vanvitelli”, Via Vivaldi, 43, 81100 Caserta, Italy; martina.slapakova@unicampania.it (M.S.); domenico.sgambati@unicampania.it (D.S.); veronica.russo1@unicampania.it (V.R.); mariangela.valletta@unicampania.it (M.V.); rosita.russo@unicampania.it (R.R.); angela.chambery@unicampania.it (A.C.); gaetano.malgieri@unicampania.it (G.M.); paolov.pedone@unicampania.it (P.V.P.); 2 Institute of Biostructures and Bioimaging, CNR, Via Pietro Castellino, 80134 Naples, Italy; luciano.pirone@cnr.it (L.P.); emilia.pedone@cnr.it (E.M.P.); 3 Department of Clinical and Experimental Medicine, University of Foggia, Viale Pinto, 1, 71122 Foggia, Italy; gianluca.dabrosca@unicampania.it; 4 Leiden Institute of Chemistry, Leiden University, 2333 CC Leiden, The Netherlands; rtdame@chem.leidenuniv.nl; 5 Centre for Microbial Cell Biology, Leiden University, 2333 CC Leiden, The Netherlands

**Keywords:** MucR, H-NS, *S. meliloti*, MucR/Ros protein family

## Abstract

Proteins of the MucR/Ros family play a crucial role in bacterial infection or symbiosis with eukaryotic hosts. MucR from *Sinorhizobium meliloti* plays a regulatory role in establishing symbiosis with the host plant, both dependent and independent of Quorum Sensing. Here, we report the first characterization of MucR isolated from *Sinorhizobium meliloti* by mass spectrometry and demonstrate that this protein forms higher-order oligomers in its native condition of expression by SEC-MALS. We show that MucR purified from *Sinorhizobium meliloti* can bind DNA and recognize the region upstream of the *ndvA* gene in EMSA, revealing that this gene is a direct target of MucR. Although MucR DNA binding activity was already described, a detailed characterization of *Sinorhizobium meliloti* DNA targets has never been reported. We, thus, analyze sequences recognized by MucR in the *rem* gene promoter, showing that this protein recognizes AT-rich sequences and does not require a consensus sequence to bind DNA. Furthermore, we investigate the dependence of MucR DNA binding on the length of DNA targets. Taken together, our studies establish MucR from *Sinorhizobium meliloti* as a member of a new family of Histone-like Nucleoid Structuring (H-NS) proteins, thus explaining the multifaceted role of this protein in many species of alpha-proteobacteria.

## 1. Introduction

The MucR/Ros protein family comprises members playing a crucial role in regulating genes involved in bacterial infection of the eukaryotic host or in bacterium–host symbiosis [1,2]. MucR/Ros proteins bind DNA through a C-terminal domain [3,4,5], which can fold around a zinc atom, thus forming a prokaryotic zinc-finger domain [6,7]. Intriguingly, some of the members of this protein family exhibit a DNA-binding domain folding without zinc in which the structural role of the metal ion is replaced by a network of hydrogen bonds [8]. The DNA binding domain present in the members of the MucR/Ros family spans from the amino acid in position 56 to the end of the proteins, where basic residues are typically located and conserved [3]. The N-terminus constitutes an oligomerization domain showing two putative alpha-helices involved in higher-order oligomer formation [9,10,11]. The structure and function of MucR and Ros have been extensively investigated. Specifically, the folding mechanism and metal coordination sphere of the prokaryotic zinc-finger present in Ros from *Agrobacterium tumefaciens* has been elucidated [6,12,13,14,15,16,17]. In *A. tumefaciens*, Ros is involved in the Horizontal Gene Transfer (HGT) of genes from the bacteria to the host plant and acts as a repressor of *virC* and *virD* operons by binding to AT-rich sequences identified in the operon promoters [1,18,19]. MucR is present in many species of alpha-proteobacteria such as *Brucella abortus, Brucella melitensis, Sinorhizobium meliloti, Sinorhizobium fredii, Caulobacter crescentus*. In *Brucella* species, the role of MucR in controlling the expression of virulence genes and of many others is well documented [2,20]. In *C. crescentus*, MucR plays a role in supporting the cell cycle transition from S-phase to G1-phase and controls transcription during G1-phase, thus coordinating the expression of target genes with cell cycle [21].

In *Rhizobia*, Ros and MucR homologs are all involved in the regulation of genes necessary for an effective symbiosis with the host plant [1,22,23,24,25,26]. In *S. meliloti*, MucR plays an essential role in the regulation of biosynthesis of the extracellular polysaccharides succinoglycan (EPS I) and galactoglycan (EPS II) [1,26,27,28,29,30,31]. These exopolysaccharides are fundamental to establishing the nitrogen-fixing symbiosis between *S. meliloti* and its host plant [1,27,31,32,33,34]. MucR regulates biosynthesis of the exopolysaccharides, likely by binding to DNA directly upstream of the *exoH* and *exoY* genes [34].

It has been proposed that *S. meliltoti* MucR plays a role in coordinating the expression of genes encoding motility factors and those necessary for EPS synthesis [29]. It was also reported that in *S. meliloti*, MucR regulates the *nod* genes whose expression is required only during symbiosis to enhance nodule formation. Furthermore, the role of MucR in regulating symbiosis with the host plant is exerted together with Quorum Sensing [35]. Nodulation also requires an appropriate response to environmental stimuli with the activation of genes involved in motility and chemotaxis, such as the *rem* gene, which is repressed by MucR in the absence of the activating environmental conditions [29].

Recently, it has been demonstrated that MucR from *B. abortus* and from *S. fredii* can form higher-order oligomers and bind DNA with a preference for AT-rich sequences without targeting any specific consensus sequence [9,10,11,36,37,38]. Oligomerization is fundamental for MucR function and proper DNA binding activity [10], and a structural model of *S. fredii* MucR high-order oligomer has been reported [11]. The similarities in functional and structural properties with Histone-like Nucleoid Structuring (H-NS) and H-NS-like proteins [39,40,41,42,43,44,45,46,47] have led to the hypothesis that MucR/Ros proteins constitute a new family of H-NS-like proteins. They might act analogous to other H-NS-like proteins in coordinating transcriptional responses to environmental conditions by their involvement in local genome organization associated with gene activity. Generally, when the expression of genes or operons is required to respond to particular environmental conditions, the binding of the H-NS proteins or the structures of nucleoids are perturbed to permit transcription [43,44,45,46,47].

Most of the studies already carried out on DNA-binding ability and structure of MucR/Ros proteins have been performed using recombinant proteins.

Here, we analyze by means of Mass Spectrometry (MS) MucR purified from *S. meliloti*, which naturally expresses this protein. Then, we show that MucR is able to form higher-order oligomers in *S. meliloti* even when the protein is not overexpressed, thus demonstrating that the quaternary structure of the protein is present in its native conditions and likely required for its function. MucR purified from *S. meliloti* can bind DNA and recognize the region upstream of the *ndvA* gene, suggesting a direct role in regulating this gene, which encodes for cyclic beta-1,2-glucan transporter playing a crucial role in nodulation [48,49]. Furthermore, we report a detailed characterization of the DNA binding activity of MucR from *S. meliloti*. We show that this protein binds AT-rich sequences preferentially without recognizing a consensus and outline the need for long targets to obtain MucR high-affinity binding.

Our findings place MucR from *S. meliloti* among Ros/MucR family members, which constitute a new family of H-NS-like proteins, thus explaining the multiple roles played by this protein identified in many alpha-proteobacteria in regulating multiple genes and operons required for successful infection or symbiosis.

## 2. Results

### 2.1. Primary Structure Characterization of MucR Purified from S. meliloti by Mass Spectrometry

A complete mass spectrometry characterization of MucR purified from *S. meliloti* was performed to obtain peptide sequences, which unequivocally identify the protein, and to investigate whether this protein undergoes post-translational modifications. The primary structure of MucR isolated from *S. meliloti* was characterized by using a strategy based on MALDI-TOF MS and high-resolution nanoLC-MS/MS analyses.

Samples of the purified MucR were digested by tripsin. Then, a peptide mapping approach was applied to analyze the digested samples by MALDI-TOF MS in both linear and reflectron positive ion modes. The accurate Mr values of peptides mapped on MucR from *S. meliloti* (AC: P55323) are reported in Table 1, and representative MS spectra are reported in Supplementary Figure S1.

In particular, an intense ion signal at m/z 7042.55 was detected mapping on the region 2–69 of MucR sequence (Supplementary Figure S1A; 2-TETSLGTSNELLVELTAEIVAAYVSNHVVPVAELPTLIADVHSALNNTTAPAPVVVPVEKPKPAVSVR-69, theoretical average [M+H^+^]^+^ mass 7042.07 Da, Δ = 0.48 ppm). This peptide accounts for about 48% of the MucR sequence. Other representative tryptic peptides identified by MALDI-TOF MS are reported in Supplementary Figure S1. Overall, by MALDI-TOF MS, a coverage of 90% of the protein sequence was obtained. Subsequently, sequencing of tryptic peptides was performed by nanoLC-ESI-MS/MS analyses on a Q-Exactive Orbitrap mass spectrometer by a data-dependent acquisition mode (Table 2).

**Figure 1:**
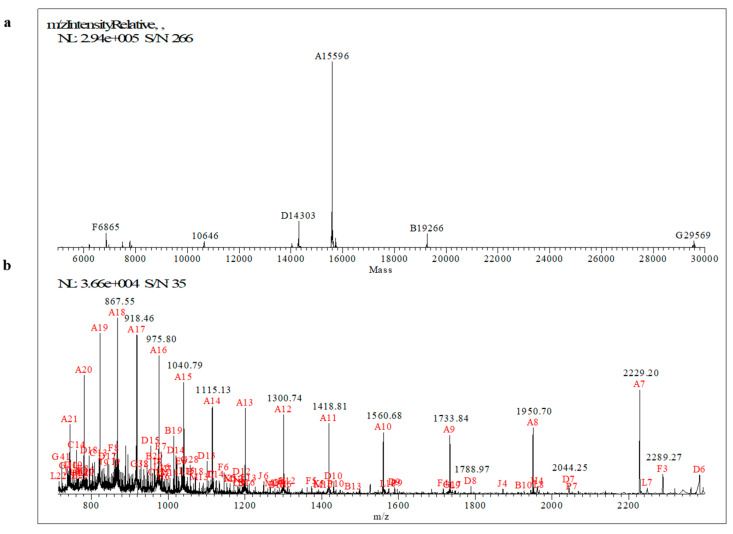
Deconvoluted (**a**) and multicharged (**b**) mass spectra of MucR determined by ESI-Orbitrap MS.

Representative MS/MS spectra of MucR are reported in Supplementary Figure S2. Since small di- and tripeptides were not detected by both MALDI-TOF and ESI-LC-MS/MS analyses, we also analyzed intact MucR to determine its whole experimental relative Mr by ESI-Orbitrap MS. The protein was eluted at 12.78 min and the mass spectra were averaged between 12.70 and 13.10 min. After deconvolution, the average multicharged spectrum gave a mass peak centered at 15596 Da (Figure 1).

This Mr differs by approximately 131 Da compared to the MucR from *S. meliloti* Mr, which corresponds to 15728 Da (MucR AC: P55323). The mass shift corresponds to the loss of a methionyl residue at the N-terminal of the protein, as also observed by MALDI-TOF analysis. No other modifications were observed on the molecular weight of MucR intact protein when the protein was purified from *S. meliloti* cultured under the planktonic condition of growth.

Furthermore, by shot-gun MS analysis of MucR purified samples we revealed the presence of low abundance peptides mapped on *S. meliloti* ribosomal proteins. This allows us to asses that purified MucR samples do not contain any other DNA-binding proteins.

### 2.2. MucR from S. meliloti Forms High-Order Oligomers

**Figure 2:**
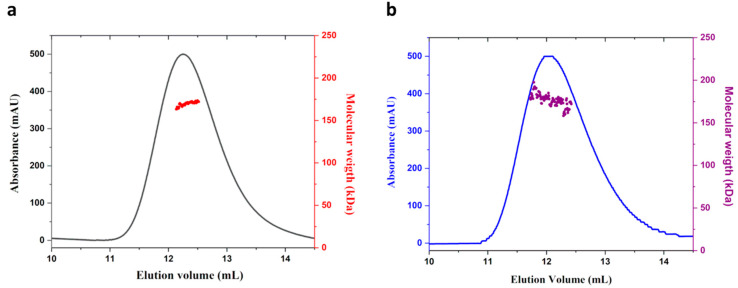
SEC-MALS analysis of (**a**) rMucR and (**b**) MucR purified from *S. meliloti.*

MucR proteins from *B. abortus* and *S. fredii* are able to form high-order oligomers [10,11]. To investigate whether *S. meliloti* MucR also exhibits oligomerization, we used *E. coli* BL21(DE3) to express and purify the recombinant *S. meliloti* MucR (rMucR). The analysis of rMucR by SEC-MALS shows that this protein forms discrete higher-order oligomers in solution with a molecular weight of 170 kDa (+/−2 kDa) (Figure 2a).

In order to verify that overexpression in *E. coli* does not affect the quaternary structure of the protein, we analyze MucR purified from *S. meliloti* (hereafter native MucR) by SEC-MALS. The result shows that the native MucR also forms higher-order oligomers with a molecular weight comparable to the one obtained for rMucR (Figure 2b).

SEC-MALS results exclude the presence of monomer and lower-order oligomers of MucR analyzed under the conditions tested. The molecular weight found corresponds to decameric MucR oligomers. Nevertheless, due to the limit of SEC-MALS, we cannot exclude the presence of higher-order oligomers formed by 9 to 12 protomers, being their molecular weight in the error of the technique used.

### 2.3. ndvA Promoter Is a Direct Target of MucR in S. meliloti

In *S. meliloti*, the *ndvA* gene encodes for cyclic beta-1,2-glucan transporter, which is fundamental for nodulation. *S. meliloti* strains bearing mutations in this gene cannot efficiently export beta-1,2-glucan in the periplasm, which is required for *S. meliloti* invasion of root nodules [48,49].

*ndvA* gene expression has been found to be regulated by MucR [29], but whether MucR interacts directly with the *ndvA* promoter has never been investigated.

**Figure 3:**
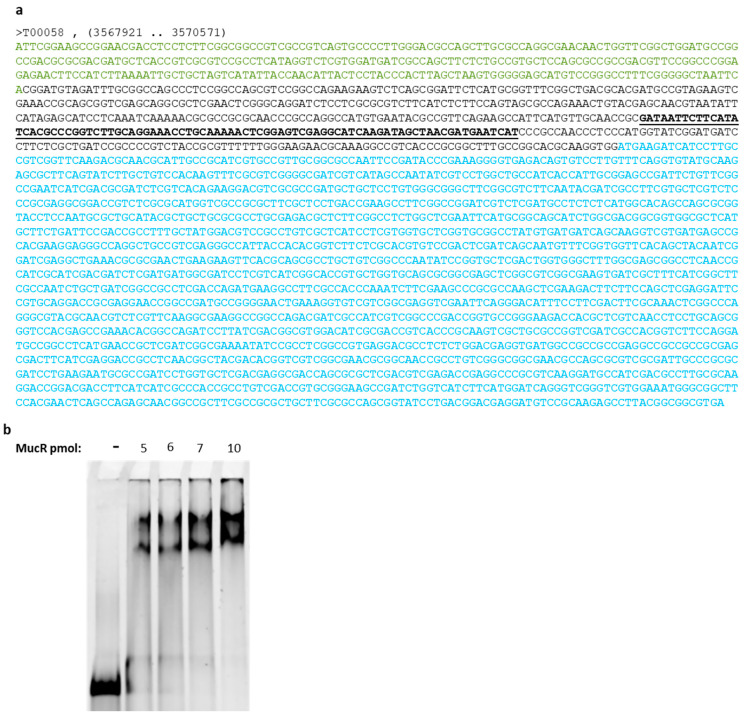
(**a**) From kegg database, the sequence of the *ndvA* gene (in blue; accession number SMc03900), the intergenic region (in black), and partial sequence (in green) of the gene (SMc03904) upstream of the intergenic region are shown. The sequence that we identify as a putative MucR target is bold and underlined; (**b**) EMSA of MucR purified from *S. meliloti* with the target identified in the intergenic region upstream of the *ndva* gene. 1.25 pmol of DNA target was used in each reaction.

We analyzed the intergenic region upstream of the *ndvA* gene, and we found that it is 44% AT-rich. Moreover, by analyzing shorter sequences in the intergenic region, we could identify a 55% AT-rich sequence (Figure 3a), which could be a typical target of MucR based on recent findings showing that MucR from different species of alpha-proteobacteria and its homologs bind preferentially to AT-rich sequences [11,36,37,38].

To investigate whether MucR could bind the intergenic region upstream of the *ndvA* gene, we used a double-stranded oligonucleotide having the 55% AT-rich sequence (Figure 3a) as a target of MucR in Electrophoretic Mobility Shift Assay (EMSA) (Figure 3b).

To perform this experiment, we used the native MucR purified from *S. meliloti* in order to verify the DNA-binding activity of this protein with a new putative target.

EMSA results in Figure 3b show that the native MucR is purified in its active fold and that the AT-rich sequence identified in the intergenic region upstream of *ndvA* is a target of this protein. The percentage of bound and free DNA are reported in Supplementary Table S1.

The results also show that MucR can form more than one complex with the chosen target. This might be due to the length of the target, which allows more than one oligomeric protein to be accommodated on the DNA and/or to obtain a different level of DNA compaction, resulting in complexes with a different electrophoretic mobility.

### 2.4. S. meliloti MucR Binds Three Different AT-Rich Sequences in the Targeted Rem Promoter without any Sequence Specificity

*rem* gene expression is necessary for motility and chemotaxis [50], and MucR is a repressor of *rem* in *S. meliloti* [29].

It was reported that MucR could bind the distal promoter of *rem* but not the proximal promoter [29].

To further investigate the DNA binding activity of MucR from *S. meliloti*, we chose to analyze the *rem* promoter sequence [50], which turned out to be 50% AT-rich. In this promoter, we identified sequences showing a different AT content, up to 70% of AT, thus constituting suitable putative MucR targets in order to analyze the effect of AT content in MucR DNA-binding activity.

**Figure 4:**
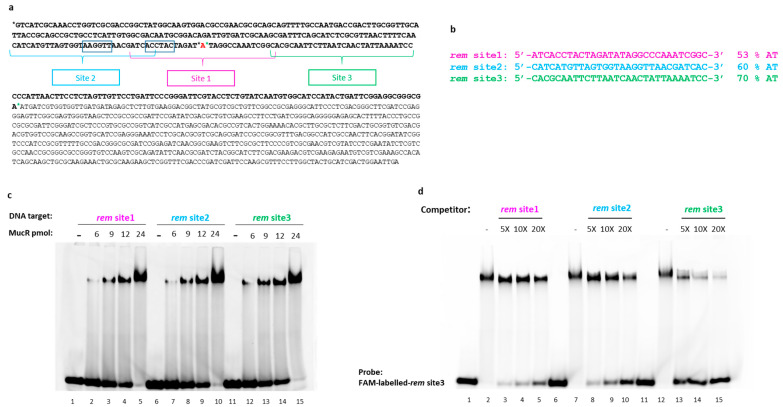
(**a**) The sequences of the *rem* promoter (in bold, upstream of the ATG start codon) and *rem* gene (starting from ATG start codon) are reported. Black asterisks and green asterisks mark the beginning and the end of the distal and the proximal promoter, respectively [29]; −35 and −10 boxes are indicated; in red is +1 position [50]. The sequence of the 30 bp oligonucleotides chosen as putative MucR targets is indicated in the promoter sequence. (**b**) Single-strand sequences of the three putative MucR targets are reported, and their AT content is indicated. (**c**) EMSA analysis of rMucR from *S. meliloti* with *rem* site1, *rem* site2, and *rem* site3. Increasing protein amounts (indicated at the top of the lanes) have been tested with 10 pmol of each double-stranded oligonucleotide. (**d**) Competition assay of *S. meliloti* MucR in complex with *rem* site3. 10 pmol of FAM-labeled double-stranded rem site3 and 20 pmol of rMucR are used to form the MucR–DNA complex. This complex was then completed by a 5-, 10-, or 20-fold excess of unlabeled *rem* site1, *rem* site2, and *rem* site3 itself (as indicated on the top of the lanes). In lanes 1, 6, and 11, only 10 pmol of the FAM-labeled-*rem* site3 was loaded; in lanes 2, 7, and 12, is shown MucR-FAM-labeled-*rem* site3 complex without adding any competitor.

On the basis of these observations, we chose three putative MucR target sites showing an increasing AT content within the same length (Figure 4a,b). The three putative targets fall into both distal and proximal promoter regions.

To test the ability of MucR to bind the putative target sites, we performed EMSAs on short dsDNA substrates corresponding to these sequences. Since the recombinant and the native MucR show the same quaternary structure (Figure 2), which is fundamental for DNA binding, and since the amount of the purified native MucR is too little for extensive DNA binding analyses, we used the recombinant MucR to perform these experiments. As shown in Figure 4c, *S. meliloti* MucR binds all three potential target sites. Our results also indicate that the proximal promoter comprises sequences that can be targeted by MucR. 

Inspired by previous results demonstrating that AT-richness positively affects DNA binding of MucR/Ros protein family members [9,10,11,36,37,38], we performed competition assays between *rem* site1, *rem* site2, and *rem* site3 (Figure 4d).

Analysis of the EMSA pattern clearly indicates that *rem* site3 better competes for binding of MucR to FAM-labeled-*rem* site3 if compared to the other two dsDNA substrates used as competitors, thus revealing that *rem* site3 is the highest affinity *S. meliloti* MucR target among the three tested. The results also show that *rem* site2 is a better competitor compared to *rem* site1, which is the least AT-rich. In fact, comparing the control experiments in which no competitor is used (Figure 4d, lanes 2, 7, and 12) to the experiments where competitors are present, it can be noticed that the intensity of the shifted band corresponding to MucR in complex with the FAM-labeled-*rem* site3 strongly decreases just adding a 5-fold excess of the unlabeled *rem* site3 as a competitor and, at the same time, the intensity of the free DNA increases (Figure 4d, lane 13). When the unlabeled competitor is *rem* site2, the decreased intensity of the band of the complex formed by MucR with the FAM-labeled-*rem* site3 and the concomitant increase of free DNA can be easily appreciated only with a 10-fold excess of unlabeled competitor (Figure 4d, lane 9), whereas a 20-fold excess of unlabeled *rem* site1 is necessary to detect a decreased intensity of the band of MucR-FAM-labeled-*rem* site3 complex and to clearly appreciate, at the same time, the increased intensity of free DNA (Figure 4d, lane 5).

The percentage of bound and free DNA detected in EMSAs shown in Figure 4c,d are reported in the Supplementary Table S2a,b, respectively.

**Figure 5:**
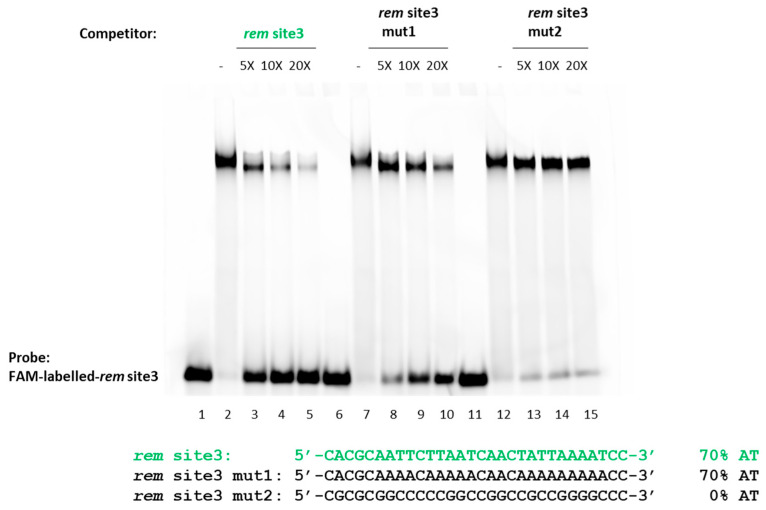
Competition assay of *S. meliloti* MucR in complex with *rem* site3. 10 pmol of FAM-labeled-*rem* site3 and 20 pmol of rMucR are used to form the MucR–DNA complex. This complex was subjected to competition with a 5-, 10-, or 20-fold excess of unlabeled *rem* site3 itself, *rem* site3 mut1, and *rem* site3 mut2 (as indicated above the lanes). In lanes 1, 6, and 11, only 10 pmol of FAM-labeled *rem* site3 was loaded; in lanes 2, 7, and 12, is shown MucR-*rem* site3 complex without adding any competitor. Sequences of only one strand of the probe site3 and of the competitors are also reported.

Previous studies showed that MucR from *B. abortus* and the homologous Ml proteins from *M. loti* [9,36,37,38] preferentially bind DNA targets with T-A steps (successive thymines and adenines in DNA sequences, interrupting long stretches of adenines) [51,52], whereas GC-rich DNA sequences are not targeted by MucR from *B. abortus* [36,37]. To investigate whether MucR from *S. meliloti* behaves in DNA binding as its homologous proteins, MucR from *B. abortus* and Ml proteins from *M. loti* [9,36,37,38] (see an alignment of homologous protein sequences in Supplementary Figure S3), we designed two variants of *rem* site3: the dsDNA substrate *rem* site3 mut1, which preserved the same AT-richness as *rem* site3 but contained several A-tracts, and *rem* site3 mut2 in which each adenine has been substituted by guanine and each thymine by a cytosine, yielding a 100% GC rich sequence (Figure 5).

We used these two variant double-stranded DNA substrates as competitors of *rem* site3 in *S. meliloti* MucR binding.

The assay reveals that *rem* site3 mut1 containing A-tracts competes with the MucR-*rem* site3 complex less effectively than the unaltered *rem* site3, indicating that the lack of T-A steps negatively affects *S. meliloti* MucR DNA-binding. The GC-rich *rem* site3 mut2 does not effectively compete with the MucR-*rem* site3 complex, indicating that GC-rich sequences are not targeted by *S. meliloti* MucR. The percentage of bound and free DNA detected in EMSA shown in Figure 5 is reported in Supplementary Table S3.

Altogether, EMSAs and competition assays show that MucR from *S. meliloti* binds preferentially more AT-rich targets containing T-A steps without any specificity, as demonstrated by the ability of MucR to recognize targets with different sequences showing no consensus. 

### 2.5. The Length of DNA Target Sites Affect S. meliloti MucR DNA Binding

SEC-MALS analysis showed that MucR from *S. meliloti* forms higher-order oligomers in solution (Figure 2). Based on this notion and the AlphaFold structure prediction of a multimer published by Shi et al. [11], we hypothesize that more than one DNA-binding domain is concurrently available for interaction with DNA target sites. It was indeed also reported that MucR from *S. fredii* can bridge DNA [11]. Bridging may occur in trans between two different DNA duplexes or in cis between two different sites on the same DNA duplex. For a protein multimer, bridging two adjacent sites (to engage two close-by DNA binding domains) on the same double-strand DNA requires a target of a sufficient length; it is expected that cooperativity arising from the simultaneous binding of two DNA binding domains enhances the effective stability of the protein–DNA complex. Based on this hypothesis and the observation that in EMSAs with 30 bp DNA substrates, the signal of free DNA was barely detectable when a protein/DNA ratio of 2.4:1 was used, we reduced the length of these substrates to 20 bp, deleting 5 bp at each extremity, yielding three shorter DNA substrates, *rem* 20 bp site1, *rem* 20 bp site2, *rem* 20 bp site3 site.

**Figure 6:**
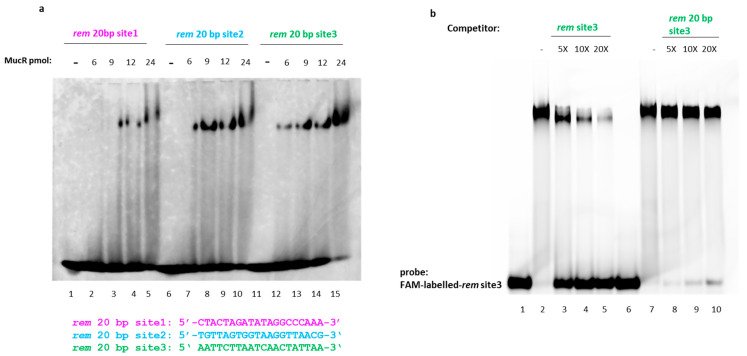
(**a**) EMSA of rMucR from *S. meliloti* with *rem* 20 bp site1, *rem* 20 bp site2, and *rem* 20 bp site3. Increasing amounts of protein (6 pmol, 9 pmol, 12 pmol, 24 pmol) were added to 10 pmol of each double-stranded DNA substrate. In lanes 1, 6, and 11, only the 10 pmol of *rem* 20 bp site1, *rem* 20 bp site2, *rem* 20 bp site3 have been loaded, respectively. (**b**) Competition assay of MucR in complex with *rem* site3. 10 pmol of FAM-labeled double-stranded *rem* site3 and 20 pmol of rMucR are used to form the MucR–DNA complex. This complex was then completed by a 5-, 10-, or 20-fold excess of unlabeled *rem* site3 and *rem* 20 bp site3 (indicated on the top of the lane). In lanes 1 and 6, only 10 pmol FAM-labeled-*rem* site3 was loaded. In lanes 2 and 7, MucR-*rem* site3 complex without adding any competitor is shown.

We used these 20 bp DNA substrates as MucR targets in EMSA, and we observed that using the same amounts of protein tested with the 30 bp DNA substrates, the band of the complex is barely detected with the *rem* 20 bp site1 and *rem* 20 bp site2 when the lowest amounts of protein are tested. The percentage of free and bound DNA detected in EMSA shown in Figure 6a is reported in Supplementary Table S4a. In order to verify the minimal length of MucR targets, we designed a shorter version of *rem* 20bp site3, deleting one base on each side of the oligonucleotide, obtaining oligonucleotides of 18 bp, 16 bp, 14 bp, and 12 bp. We tested these short DNA substrates as targets of MucR by EMSAs. The results show that the minimal length of DNA to detect MucR binding in EMSA is 18 bp (Supplementary Figure S4).

In EMSA using *rem* 20 bp site3, which showed to be the best 20 bp target, free DNA is still clearly detectable at a protein/DNA ratio of 2.4:1 (Figure 6a).

To confirm *rem* 20bp site3 is a worse MucR target than *rem* 30 bp site3, we performed a competition assay in which the complex formed by MucR with *rem* site3 (30 bp) is competed by itself and by *rem* 20 bp site3 (Figure 6b).

Clearly, the *rem* 20 bp site3 does not effectively compete with MucR binding to *rem* site3, which is 30 bp long, confirming that the length of the DNA target plays a fundamental role in increasing the apparent affinity of MucR DNA binding. Supplementary Table S4 reports the percentage of bound and free DNA detected in EMSAs shown in Figure 6. A molecular docking model of Ros87-DNA interaction provided by Russo et al. [4] offers insights into the molecular DNA recognition process of the prokaryotic zinc finger domains. The model indicates that the interaction involves the first α-helix of the zinc finger domain and additional residues located in the basic regions flanking it, with the C-terminal tail that is wrapped around the DNA. As a result, the prokaryotic zinc finger domain binds an extended recognition site that spans at least 15 base pairs. To further verify our hypothesis, we have generated a model of the MucR–DNA complex by using a dimeric structure of MucR generated by AlphaFold and a model structure of the 20 bp/30 bp DNA probe in the B-form conformation, generated as reported in the Section 4.

**Figure 7:**
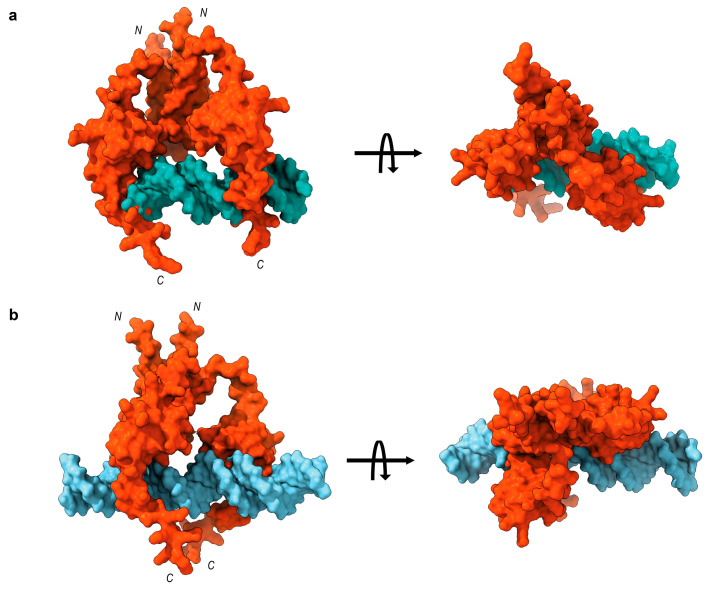
MucR–DNA complex: pyDockDNA best-ranking binding poses of (**a**) MucR-Dimer/20 bp DNA probe and (**b**) MucR-Dimer/30 bp DNA probe.

Our model has been merely built to verify the spatial possibility of binding a 20 bp/30 bp DNA probe to more than one MucR DNA-binding domain, and it is not expected to define the protein–DNA contacts stabilizing such interaction. Nonetheless, in accordance with the previously published study [4], the models clearly evidence the chance for the longer DNA structure to nicely accommodate the dimeric form of the protein, suggesting the possibility of the two DNA-binding motifs and their C-terminal tails to interact with the 30 bp DNA probe. On the other hand, the model built using the shorter DNA structure indicates the 20 bp DNA probe as too short to accommodate two DNA-binding domains along with both their C-terminal tails, giving a possible explanation for the lower affinity shown by the protein toward this binding probe (Figure 7a,b).

The results obtained support the hypothesis that the length of the DNA targets is a crucial factor in achieving MucR–DNA high affinity binding.

## 3. Discussion

The Ros/MucR protein family comprises members having a role in symbiosis or in the infection process of the eukaryotic host. In particular, *Rhizobia* species induce the formation of nodules, which are new organs on legume roots that can be colonized by symbiotic bacteria [53]. 

In this study, we report the first structural characterization of the native MucR purified from *S. meliloti* by mass spectrometry and SEC-MALS techniques. We show that this protein does not undergo post-translational modifications and forms higher-order oligomers in *S. meliloti*, thus providing the first evidence that MucR oligomers are formed in the bacterial species naturally expressing this protein and suggesting that oligomerization is necessary for MucR function in *S. meliloti*. Post-translational modifications have been recently detected in some nucleoid-associated proteins (NAPs), which have the role of compacting genomic DNA in bacteria, even if the role of such modifications is still under investigation [54]. In this study, we purified MucR from *S. meliloti* grown in LB under planktonic conditions; thus, we cannot exclude that MucR can undergo post-translation modifications when *S. meliloti* bacterial cells live in biofilm, in symbiosis with host plant and/or under stress conditions.

Furthermore, we show that MucR isolated from *S. meliloti* can bind the intergenic region upstream of the *ndvA* gene, suggesting that the regulatory function of MucR in the expression of this gene encoding for cyclic beta-1,2-glucan transporter [29,48], can be due to a direct interaction of the protein with the regulatory region of *ndvA*.

Moreover, we report a detailed analysis of the *S. meliloti* MucR DNA-binding activity, showing that this protein binds AT-rich DNA targets containing T-A steps as other members of the MucR/Ros family [9,11,36,37] and without the need to recognize a consensus sequence.

Our data also reveal that *S. meliloti* MucR binds DNA targets of 30 bp with high affinity, whereas the binding affinity decreases when 20 bp targets are used.

The data reported here are in line with the definition of MucR from *S. meliloti* as a new type of H-NS. Proteins structuring the nucleoid in bacteria bind AT-rich DNA as oligomers capable of bridging DNA to achieve compaction of the nucleoid [39,40,41,42,43,44,45,46,47,55,56]. Data here reported suggest that the oligomeric structure of MucR requires long targets so that more than one DNA-binding domain of the oligomers can contact the nucleic acid. Based on the AlphaFold structural prediction published by Shi et al. [11], another possibility is that long DNA targets more easily allow the MucR oligomers to bridge different sites on the same double-strand DNA, thus improving the stability of MucR with target sites. Finally, our observations about the importance of the length of DNA targets in MucR binding also provide an explanation of data presented in different studies which reported that long degenerated target sites are involved in the interaction of MucR and its homologous proteins with DNA [19,21,29,30,34].

Overall, we demonstrate that MucR from *S. meliloti* has a DNA binding activity very similar to that reported for other members of the Ros/MucR family, strengthening the hypothesis that this family constitutes a new type of H-NS-like proteins and underlying the importance of having more structural data concerning the MucR oligomers and in complex with DNA.

## 4. Materials and Methods

### 4.1. Protein Expression and Purification

The coding sequence for MucR from *S. meliloti* was amplified by PCR using genomic DNA from *S. meliloti* 1021 as a template. Primers were designed based on the mucR gene sequence available in the NCBI database (NCBI Reference Sequence: NC_020528.1). Sequences of the primers used are the following: forward primer: 5′ ACTCTACATATGACAGAGACTTCGCTCGGT ACGAGC 3′; reverse primer: 5′ GTCCACTCGAGTCACTTGCCGCGACGCTTCCGACG 3′.

The PCR amplicon was gel purified, digested with *NdeI* and *XhoI* enzymes (New England Biolabs, Ipswich, MA, USA), and used for cloning into the pET22b+ vector digested with the same enzymes. Clones positive for the presence of the insert were sequenced using the Sanger method.

Expression of the recombinant MucR was carried out by transforming *E. coli* Bl21 (DE3) with Smel_MucR_pet22b+. Protein expression was induced at an optical density of the culture at 600 nm of 0.450. Then, 1 mM IPTG and 100 μM of zinc sulfate were added to the culture and incubated at 28 °C until reaching the optical density value of 0.8 at 600 nm.

*S. meliloti* 1021 was cultured in LB with the addition of 100 μg/mL of streptomicyn at 30 °C until reaching the optical density value of 1.2 at 600 nm.

Purification of the recombinant MucR from *E. coli* Bl21 (DE3) and the naturally expressed MucR from *S. meliloti* 1021 was carried out as previously described [3].

Briefly, bacterial cells were harvested by centrifugation, resuspended in Tris 25 mM pH 7, and sonicated on ice. Clear lysates were obtained by centrifugation for 30′ at 27,450 rcf. Proteins were purified from the soluble fraction using cation exchange chromatography and the following step of size exclusion chromatography. Samples obtained after the last step of purification of MucR from *S. meliloti* 1021 were analyzed by mass spectrometry to check the presence of MucR protein.

### 4.2. High-Resolution NanoLC–Tandem Mass Spectrometry

Aliquots of protein samples (50 µg) were reduced with 20 mM dithiothreitol (DTT, 5 min at 95 °C) and alkylated with 20 mM iodoacetamide (IAA, 30 min, in the dark, at room temperature). Enzymatic hydrolyses were performed on reduced and alkylated samples by adding TPCK-treated trypsin with an enzyme/substrate (E/S) ratio of 1:200 (*w*/*w*) for 3 h, 1:100 for 16 h, and 1:50 for 4 h at 37 °C. Mass spectrometry analyses on tryptic samples (500 fmol) were performed on a Q-Exactive Orbitrap mass spectrometer equipped with an EASY-Spray nano-electrospray ion source (Thermo Fisher Scientific, Bremen, Germany) and coupled with a Thermo Scientific Dionex UltiMate 3000 RSLCnano system (Thermo Fisher Scientific, Rockford, IL, USA). The solvent composition was 0.1% formic acid in water (solvent A) and 0.1% formic acid in acetonitrile (solvent B). Peptides were loaded on a trapping PepMap™100 µCartridge Column C18 (300 µm × 0.5 cm, 5 µm, 100 angstroms) and desalted with solvent A for 3 min at a flow rate of 10 µL/min. After trapping, eluted peptides were separated on an EASY-Spray analytical column (50 cm × 75 µm ID PepMap RSLC C18, 3 μm, 100 angstroms) and heated to 35 °C at a flow rate of 300 nL/min using the following gradient: 4% B for 3 min, from 4% to 55% B in 60 min, from 55% to 70% B in 10 min, and from 70% to 95% B in 2 min. Eluting peptides were analyzed on the Q-Exactive mass spectrometer operating in positive polarity mode with a capillary temperature of 280 °C and a potential of 1.9 kV applied to the capillary probe. Full MS survey scan resolution was set to 70,000 with an automatic gain control (AGC) target value of 3 × 106 for a scan range of 375–1500 *m*/*z* and maximum ion injection time (IT) of 100 ms. The mass (*m*/*z*) 445.12003 was used as lock mass. A data-dependent top 5 method was operated, during which higher-energy collisional dissociation (HCD) spectra were obtained at 17,500 MS2 resolution with AGC target of 1 × 10^5^ for a scan range of 200–2000 *m*/*z*, maximum IT of 55 ms, 2 *m*/*z* isolation width, and normalized collisional energy (NCE) of 27. Precursor ions targeted for HCD were dynamically excluded for 15 s. Full scans and Orbitrap MS/MS scans were acquired in profile mode, whereas ion trap mass spectra were acquired in centroid mode. Charge state recognition was enabled by excluding unassigned charge states. The acquired raw files were analyzed with Proteome Discoverer 2.4 software (Thermo Fisher Scientific, Rockford, IL, USA) using the SEQUEST HT search engine. The HCD MS/MS spectra were searched against the whole UniProt_SwissProt KB database, assuming trypsin (Full) as a digestion enzyme with two allowed numbers of missed cleavage sites. The mass tolerances were set to 10 ppm and 0.02 Da for precursor and fragment ions, respectively. Oxidation of methionine (+15.995 Da) was set as dynamic modification, and carbamidomethylation of cysteine (+57.021 Da) as static modification [57].

### 4.3. MALDI-TOF MS Analysis of MucR Peptides

Peptides obtained by tryptic digestion, as described above, were analyzed with a MALDI-TOF mass spectrometer (Waters Micromass Co., Manchester, UK) [58]. For MALDI-TOF MS analysis, 1 μL of peptide mixture or single peptide solution was mixed with 1 μL of saturated α-cyano-4-hydroxycinnamic acid matrix solution (10 mg/mL in acetonitrile: 0.1% TFA in water (1:1; *v*/*v*). Thus, a droplet of the resulting mixture (1 μL) was placed on the mass spectrometer’s sample target and dried at room temperature. Once the liquid was completely evaporated, samples were loaded into the mass spectrometer and analyzed in positive ion mode. In reflection mode, the instrument was externally calibrated using a tryptic alcohol dehydrogenase digest (Waters). For linear mode, a 4-point external calibration was applied using an appropriate mixture (10 pmol/μL) of insulin, cytochrome C, horse Mb, and trypsinogen as standard proteins (Sigma-Aldrich). A mass accuracy near the nominal (50 and 300 ppm in reflectron and linear modes, respectively) was achieved for each standard. All spectra were processed and analyzed by using the Mass Lynx 4.1 software.

### 4.4. Mass Spectrometry Analysis of Intact Protein

MucR from *S. meliloti* 1021 was analyzed by LC-MS using a Thermo Fisher system equipped with a binary pump, an automated autosampler, a multi-wavelength Diode Array detector (Ultimate 3000), and an Orbitrap high-resolution mass spectrometer (Q-Exactive Plus, max resolution 280,000). For the analysis, an RP-4 LUNA 50 × 2 mm ID column was used, equilibrated at 25% solvent B (acetonitrile, 0.05% TFA) over solvent A (H_2_O, 0.05% TFA). The protein (around 300 ng) was eluted with a 15 min gradient from 25% to 70% solvent B in 15 min at 0.2 mL/min. For the mass spectrometry analysis, the Q-Exactive MS was operated in the intact protein (C-Trap pressure at 1.0) positive ion mode with the source maintained at 3 kV and 300 °C. The desolvating N2 gas was kept at 25 L/min and at 50 °C. Spectra were continuously recorded between 600 and 2400 *m*/*z* at a resolution of 17,500. The AGC target was set to 1 × 10^6^. The S-lens RF level was set at 80. One microscan was collected for every spectrum with a maximum injection time of 200 ms. Multicharged spectra were deconvoluted using MagTran Version 1.0.2.

### 4.5. SEC-MALS

In order to determine the molecular weight of the protein object of this study, a MiniDAWN Treos spectrometer (Wyatt Instrument Technology Corp., Goleta, CA, USA) equipped with a laser operating at 658 nm was used and connected on-line to a size-exclusion chromatography column. Samples at a concentration of 1 mg/mL were loaded onto a Superdex 200 column (10 × 30 cm, GEHealthcare) equilibrated in the same buffer used for the final purification procedure and connected to a triple-angle light scattering detector equipped with a QELS (Quasi-Elastic Light Scattering) module. A constant flow rate of 0.5 mL/min was applied. Elution profiles were detected by a Shodex interferometric refractometer and a mini Dawn TREOS light scattering system. The Astra 5.3.4.14 software (Wyatt Technology, Santa Barbara, CA, USA) was used to analyze data. Duplicates of each experiment were carried out.

### 4.6. EMSA

EMSA experiments were carried out as previously reported [59].

Briefly, several amounts of the recombinant MucR purified from *E. coli* Bl21 (DE3) and of the naturally expressed MucR from *S. meliloti* 1021 were mixed with the DNA target sites tested in binding buffer (25 mM HEPES pH 7.9, 50 mM KCl, 6.25 mM MgCl_2_, 5% glycerol). Samples were incubated 10 min on ice and loaded in a 5% polyacrylamide gel. Electrophoresis was performed in 0.5× TBE and run at room temperature for 70 min at 200 V. Gels were stained for 20 min using Diamond™ Nucleic Acid Dye (Promega, Singapore) following the manufacturer’s instructions and imaged by Typhoon Trio+ scanner (GE Healthcare, Chicago, IL, USA).

For competition assays, competitors were added to the reaction mixture, prepared as described above, after proteins were incubated 10 min on ice with the FAM-labeled double-stranded oligonucleotides. After adding the competitors, samples were incubated for a further 10 min on ice and then loaded in a 5% polyacrylamide gel. Images were acquired by Typhoon Trio+ scanner (GE Healthcare) on the base of the FAM-labeled DNA fluorescence.

Protein amounts used, sequences, and amounts of double-stranded DNA targets are indicated in figures and figure legends.

### 4.7. Molecular Docking

The three-dimensional structure of the MucR dimer was generated using the AlphaFold2 Multimer [60] algorithm. The best ranking model was chosen and then prepared for the docking simulation using the tools provided by Samson software (https://www.samson-connect.net) (OneAngstrom, Grenoble, France). The 20 bp and 30 bp DNA, B-form, double helix models were built by means of ChimeraX software (version 1.6.1 https://www.cgl.ucsf.edu/chimerax/) [61] using the nucleotide sequences previously reported in EMSA experiments. Docking simulations were performed using the rigid-body method of the pyDockDNA server [62], and the peptide contact constraints were forced according to ref [4] for both monomers of each model. The best-ranking docking pose for each complex (20 bp-DNA/MucR-dimer and 30 bp-DNA/MucR-dimer) was selected on the basis of the lowest pyDockDNA binding energy, and the software ChimeraX and pyMol were used to visualize and depict them.

## Figures and Tables

**Table 1:** Tryptic peptides obtained by MALDI-TOF MS analysis of MucR. Sequence position, experimental and theoretical molecular weights of peptides (MH^+^), mass accuracies together with missed cleavage sites, and modifications are reported. CAM = carbamidomethyl cysteine. Average Mr values are shown for molecular weight values above 3 k.

Tryptic Peptide	Sequence Position	Exp Mr [M+H^+^]^+^	Th Mr [M+H^+^]^+^	Δ (Da)	Missed Cleavages	Modifications
T1	2–69	7042.55	7042.07	0.48		Methionyl residue loss
T2	71–87	1957.84	1957.86	0.02		
T3	70–87	2085.90	2085.96	0.06	K70	CAM (C79, C82)
T4	92–105	1763.80	1763.83	0.03		
T5	106–125	2278.94	2279.08	0.14	K107	
T6	131–137	790.26	790.39	0.13		
T7	131–138	946.44	946.49	0.05		

**Table 2:** Amino acid sequences of tryptic peptides from MucR obtained by high-resolution nanoLC-Tandem mass spectrometry. Sequence, modifications number of missed cleavage sites (MC), experimental masses of precursor ions, charge state, experimental and theoretical molecular weights of peptides (MH^+^) together with mass accuracies and retention times are reported. CAM = carbamidomethyl cysteine.

Sequence	Modifications	MC	Charge	*m*/*z* (Da)	Exp Mr [M+H^+^]^+^	Th Mr [M+H^+^]^+^	Δ (ppm)	RT (min)
DKWDLPADYPMVAPAYAEAR		1	3	760.364	2279.078	2279.080	−0.84	44.3
KSVQDDQITcLEcGGTFK	CAM (C10, C13)	1	3	695.990	2085.957	2085.958	−0.52	37.0
SVQDDQITcLEcGGTFK	CAM (C9, C12)	0	2	979.434	1957.861	1957.863	−1.08	40.0
SVQDDQITcLEcGGTFK	CAM (C9, C12)	0	3	653.292	1957.862	1957.863	−0.53	40.0
EMGLGQR		0	2	395.697	790.387	790.388	−0.44	31.3

## Data Availability

The data presented in this study are all available in this article “MucR from *Sinorhizobium meliloti*: new insights into its DNA targets and its ability to oligomerize” and in its supplementary material.

## Supplementary Materials

The supporting information can be downloaded at: https://www.mdpi.com/article/10.3390/ijms241914702/s1.
