# Supplementary material for: MucR from Sinorhizobium meliloti: New Insights into Its DNA Targets and Its Ability to Oligomerize
Source: Int J Mol Sci. 2023 Sep 29;24(19):14702. doi: 10.3390/ijms241914702 (PMC10572780; doi:10.3390/ijms241914702)
Supplement: Supplementary file 1 [file ijms-24-14702-s001.zip › ijms-2601346-supplementary.pptx]

## Slide 1
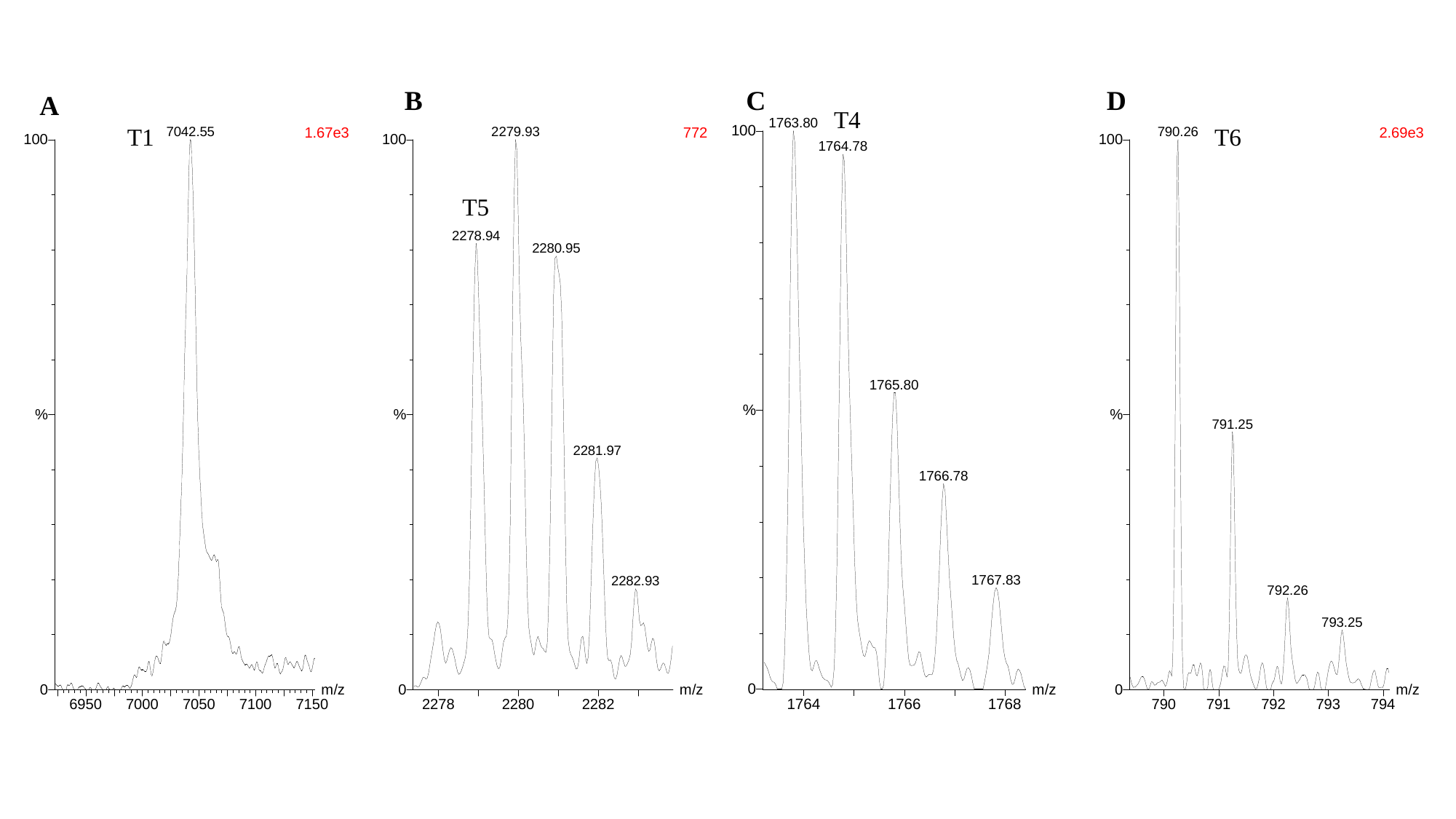

B
C
D
A
T4
T1
T6
T5

## Slide 2
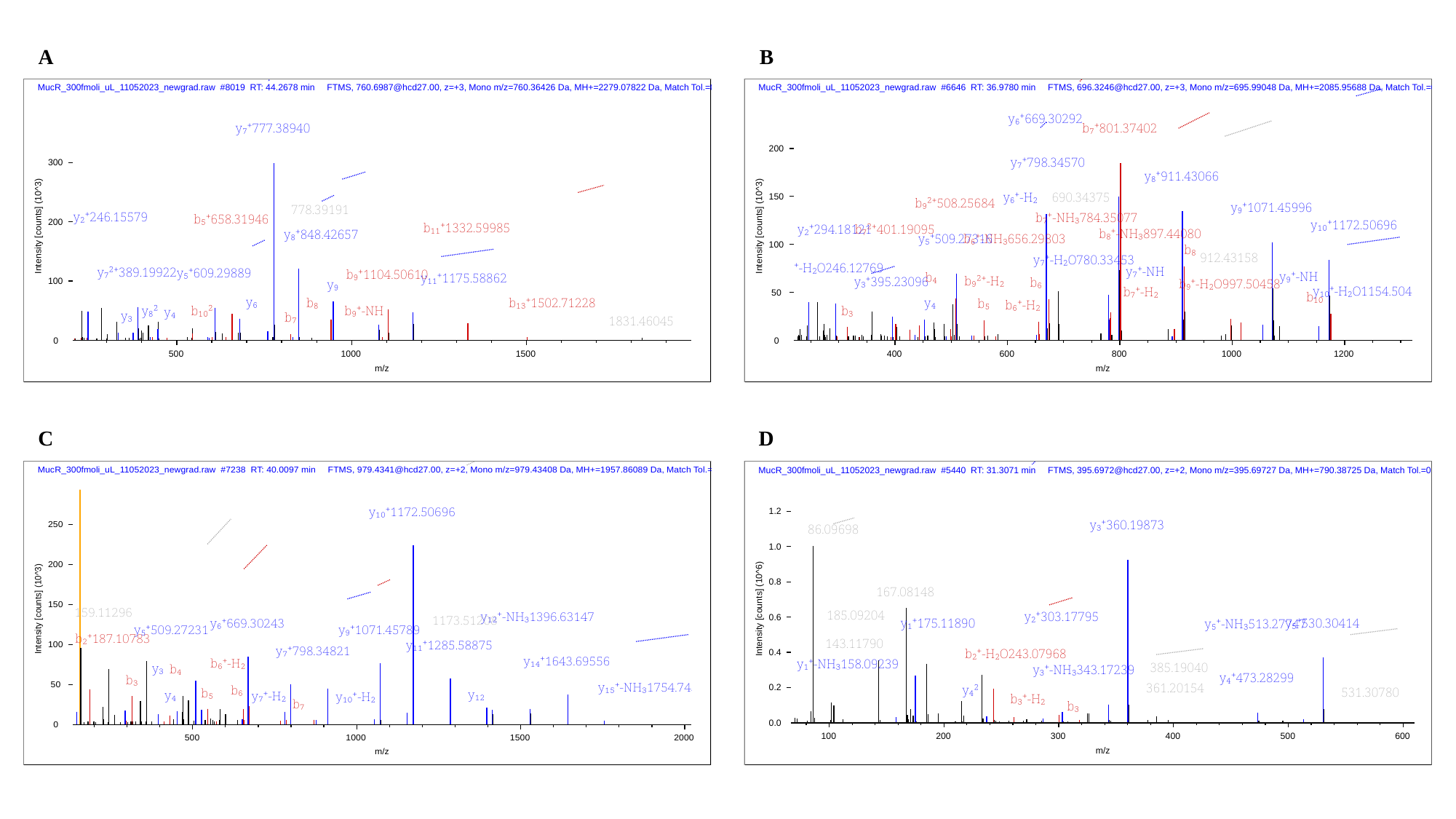

A
B
C
D

## Slide 3
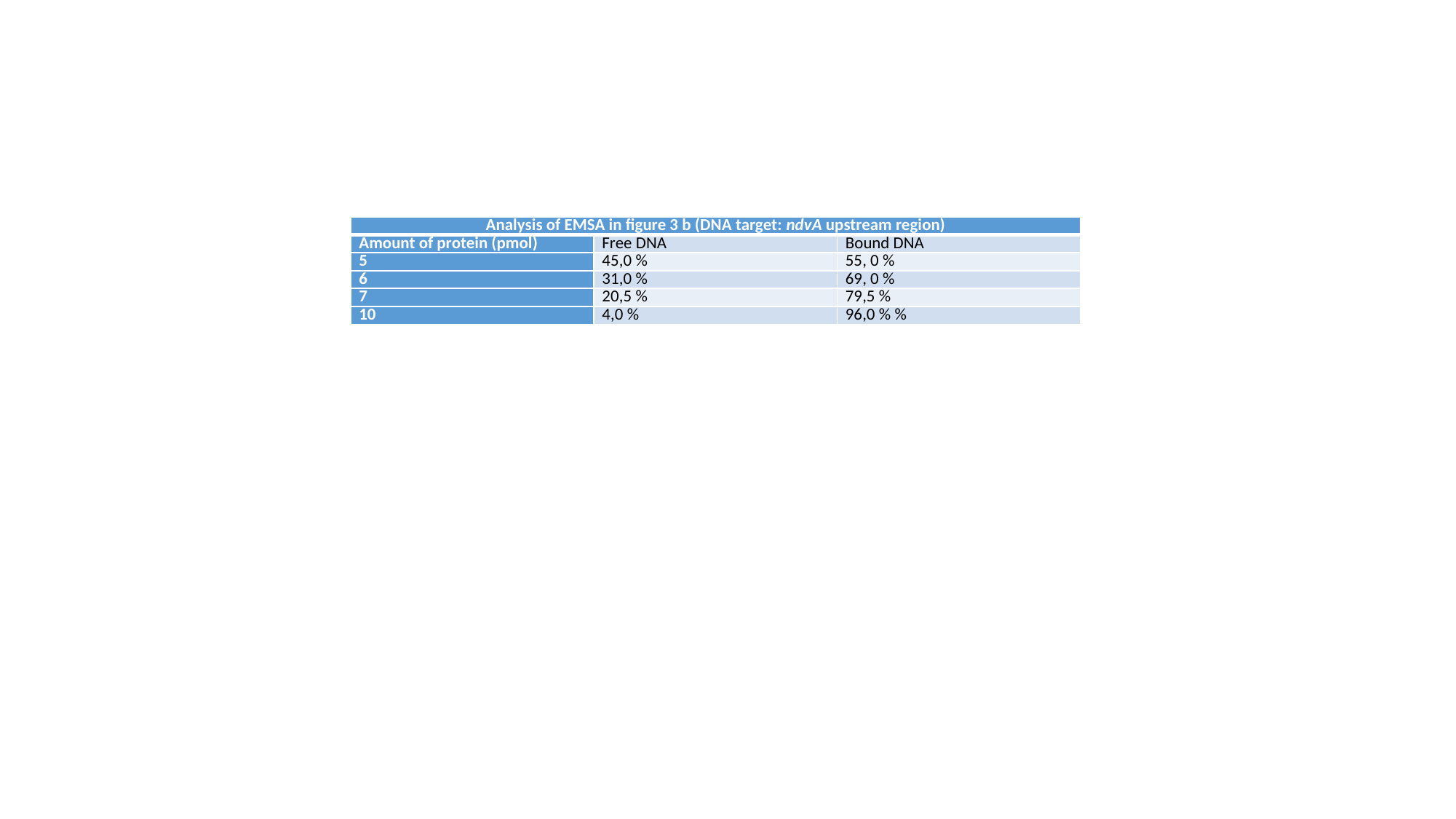

| Analysis of EMSA in figure 3 b (DNA target: ndvA upstream region) | | |
| --- | --- | --- |
| Amount of protein (pmol) | Free DNA | Bound DNA |
| 5 | 45,0 % | 55, 0 % |
| 6 | 31,0 % | 69, 0 % |
| 7 | 20,5 % | 79,5 % |
| 10 | 4,0 % | 96,0 % % |

## Slide 4
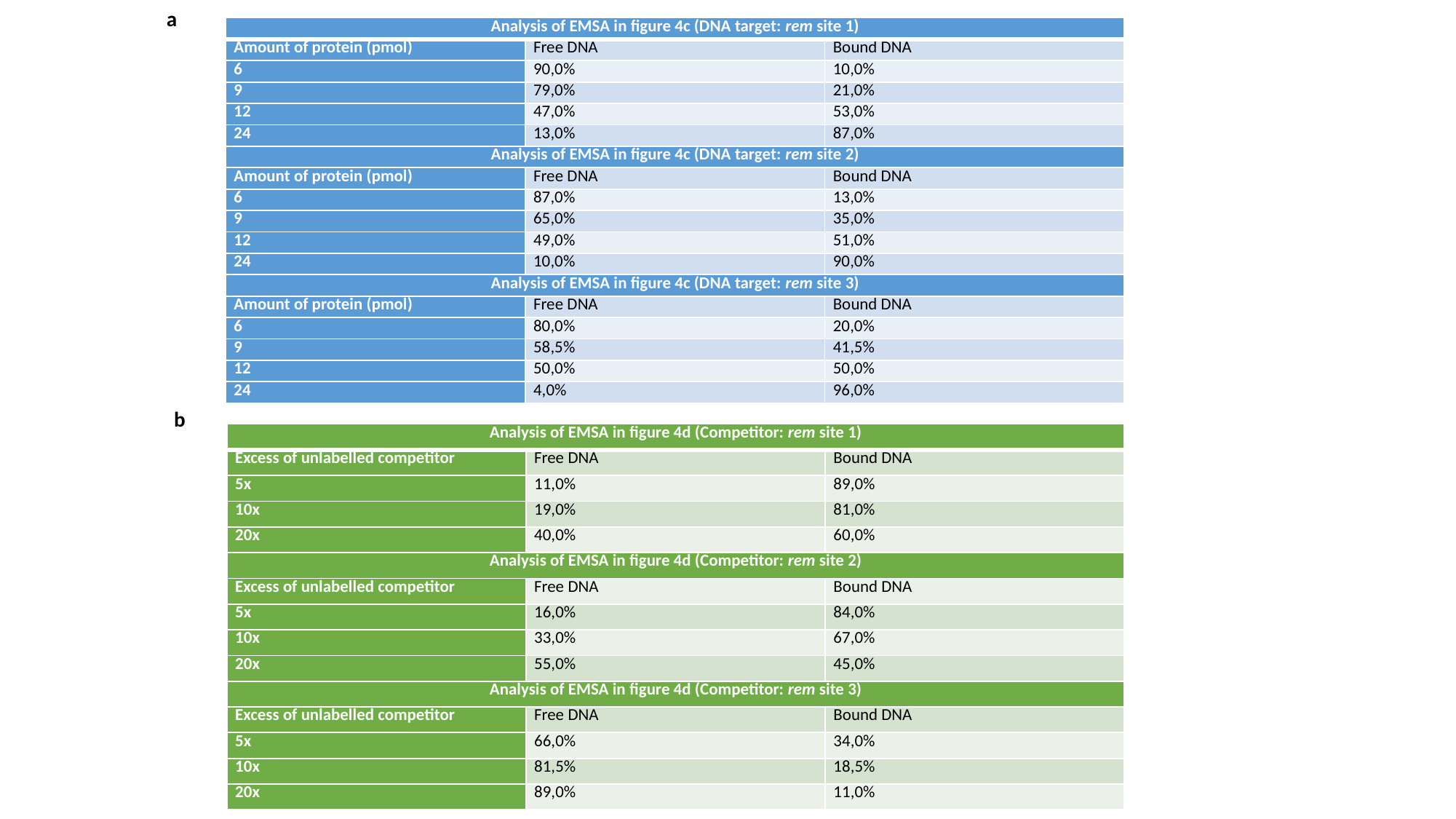

a
| Analysis of EMSA in figure 4c (DNA target: rem site 1) | | |
| --- | --- | --- |
| Amount of protein (pmol) | Free DNA | Bound DNA |
| 6 | 90,0% | 10,0% |
| 9 | 79,0% | 21,0% |
| 12 | 47,0% | 53,0% |
| 24 | 13,0% | 87,0% |
| Analysis of EMSA in figure 4c (DNA target: rem site 2) | | |
| Amount of protein (pmol) | Free DNA | Bound DNA |
| 6 | 87,0% | 13,0% |
| 9 | 65,0% | 35,0% |
| 12 | 49,0% | 51,0% |
| 24 | 10,0% | 90,0% |
| Analysis of EMSA in figure 4c (DNA target: rem site 3) | | |
| Amount of protein (pmol) | Free DNA | Bound DNA |
| 6 | 80,0% | 20,0% |
| 9 | 58,5% | 41,5% |
| 12 | 50,0% | 50,0% |
| 24 | 4,0% | 96,0% |
b
| Analysis of EMSA in figure 4d (Competitor: rem site 1) | | |
| --- | --- | --- |
| Excess of unlabelled competitor | Free DNA | Bound DNA |
| 5x | 11,0% | 89,0% |
| 10x | 19,0% | 81,0% |
| 20x | 40,0% | 60,0% |
| Analysis of EMSA in figure 4d (Competitor: rem site 2) | | |
| Excess of unlabelled competitor | Free DNA | Bound DNA |
| 5x | 16,0% | 84,0% |
| 10x | 33,0% | 67,0% |
| 20x | 55,0% | 45,0% |
| Analysis of EMSA in figure 4d (Competitor: rem site 3) | | |
| Excess of unlabelled competitor | Free DNA | Bound DNA |
| 5x | 66,0% | 34,0% |
| 10x | 81,5% | 18,5% |
| 20x | 89,0% | 11,0% |

## Slide 5
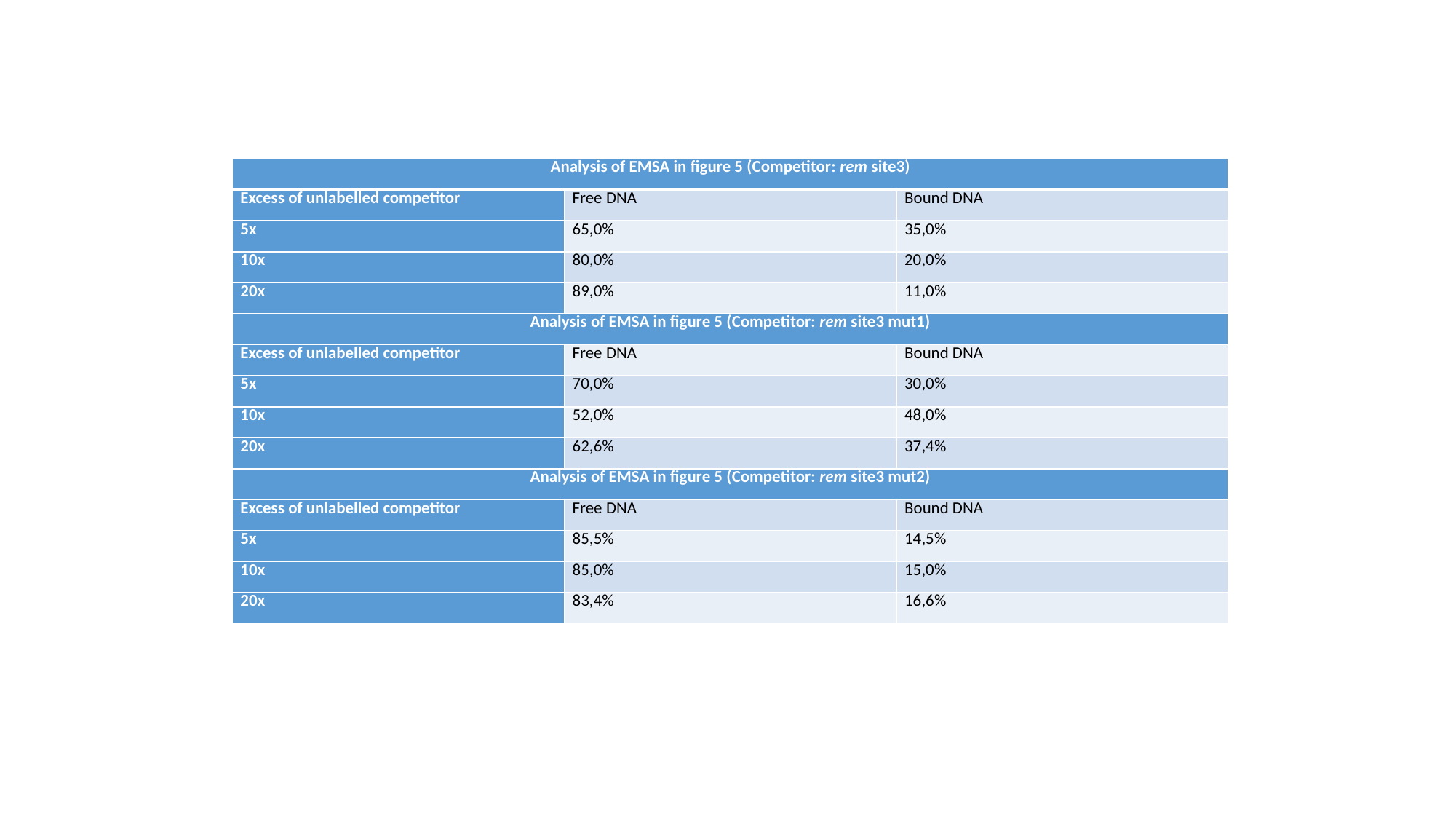

| Analysis of EMSA in figure 5 (Competitor: rem site3) | | |
| --- | --- | --- |
| Excess of unlabelled competitor | Free DNA | Bound DNA |
| 5x | 65,0% | 35,0% |
| 10x | 80,0% | 20,0% |
| 20x | 89,0% | 11,0% |
| Analysis of EMSA in figure 5 (Competitor: rem site3 mut1) | | |
| Excess of unlabelled competitor | Free DNA | Bound DNA |
| 5x | 70,0% | 30,0% |
| 10x | 52,0% | 48,0% |
| 20x | 62,6% | 37,4% |
| Analysis of EMSA in figure 5 (Competitor: rem site3 mut2) | | |
| Excess of unlabelled competitor | Free DNA | Bound DNA |
| 5x | 85,5% | 14,5% |
| 10x | 85,0% | 15,0% |
| 20x | 83,4% | 16,6% |

## Slide 6
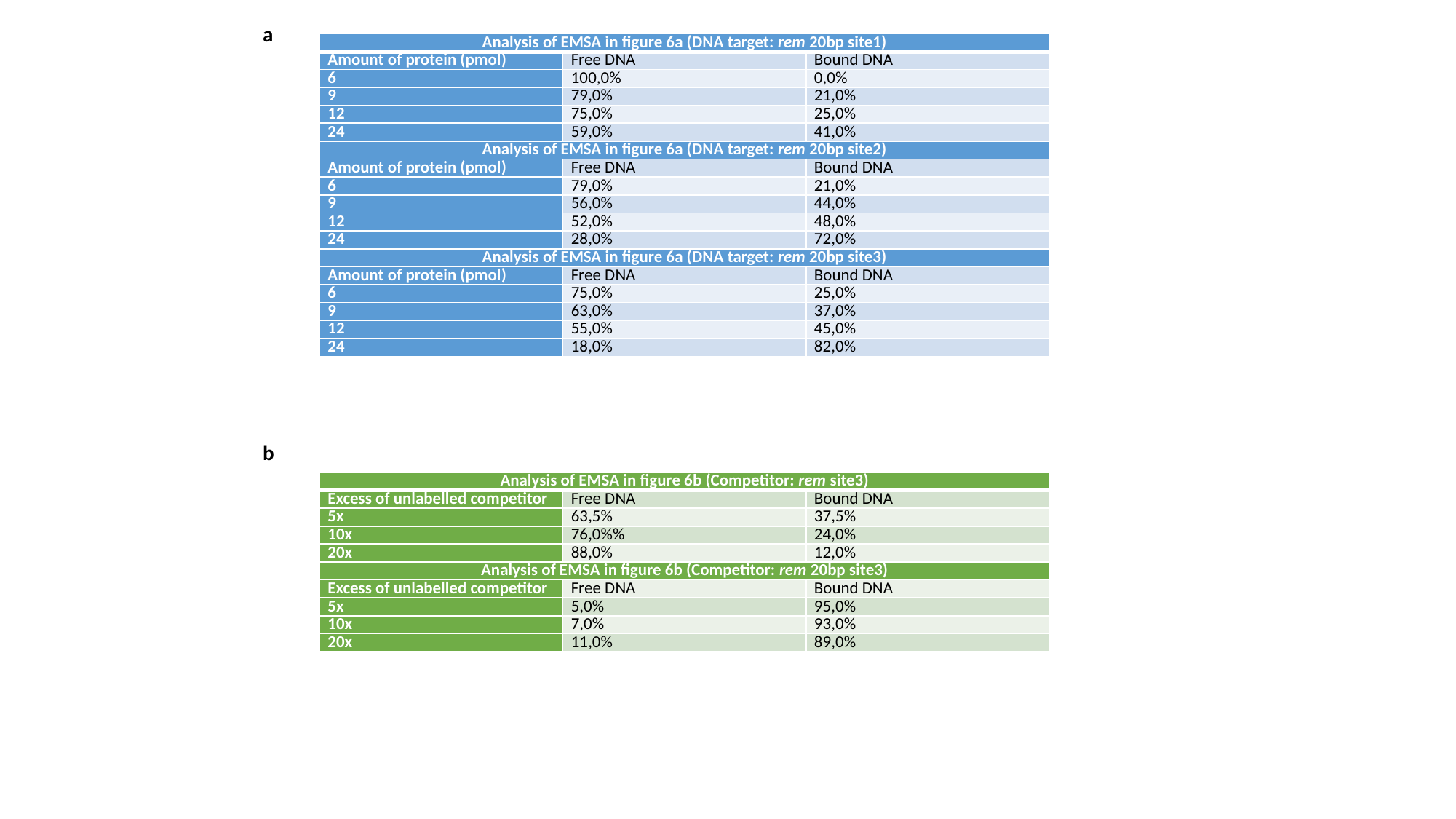

a
| Analysis of EMSA in figure 6a (DNA target: rem 20bp site1) | | |
| --- | --- | --- |
| Amount of protein (pmol) | Free DNA | Bound DNA |
| 6 | 100,0% | 0,0% |
| 9 | 79,0% | 21,0% |
| 12 | 75,0% | 25,0% |
| 24 | 59,0% | 41,0% |
| Analysis of EMSA in figure 6a (DNA target: rem 20bp site2) | | |
| Amount of protein (pmol) | Free DNA | Bound DNA |
| 6 | 79,0% | 21,0% |
| 9 | 56,0% | 44,0% |
| 12 | 52,0% | 48,0% |
| 24 | 28,0% | 72,0% |
| Analysis of EMSA in figure 6a (DNA target: rem 20bp site3) | | |
| Amount of protein (pmol) | Free DNA | Bound DNA |
| 6 | 75,0% | 25,0% |
| 9 | 63,0% | 37,0% |
| 12 | 55,0% | 45,0% |
| 24 | 18,0% | 82,0% |
b
| Analysis of EMSA in figure 6b (Competitor: rem site3) | | |
| --- | --- | --- |
| Excess of unlabelled competitor | Free DNA | Bound DNA |
| 5x | 63,5% | 37,5% |
| 10x | 76,0%% | 24,0% |
| 20x | 88,0% | 12,0% |
| Analysis of EMSA in figure 6b (Competitor: rem 20bp site3) | | |
| Excess of unlabelled competitor | Free DNA | Bound DNA |
| 5x | 5,0% | 95,0% |
| 10x | 7,0% | 93,0% |
| 20x | 11,0% | 89,0% |

## Slide 7
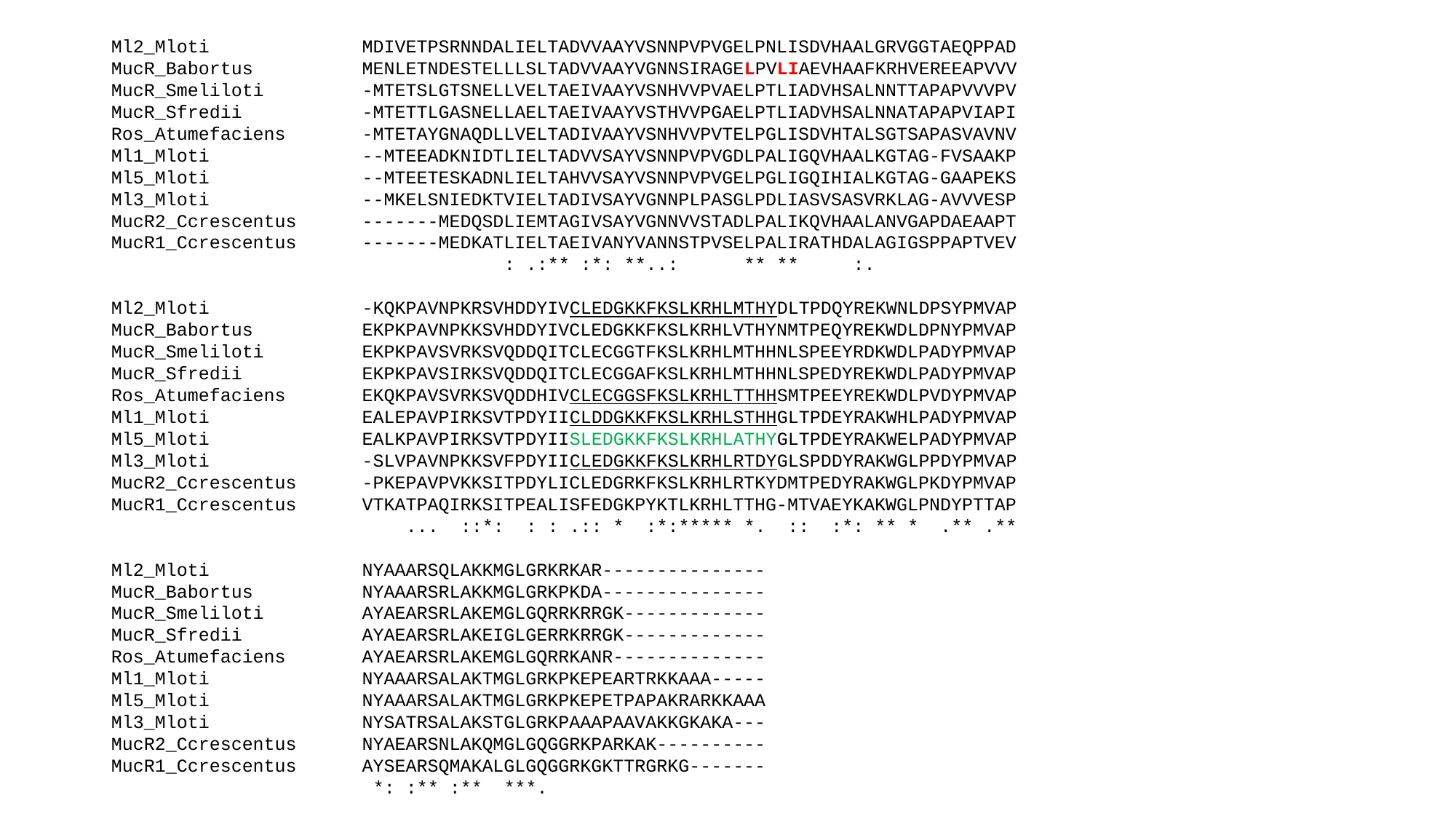

Ml2_Mloti MDIVETPSRNNDALIELTADVVAAYVSNNPVPVGELPNLISDVHAALGRVGGTAEQPPAD
MucR_Babortus MENLETNDESTELLLSLTADVVAAYVGNNSIRAGELPVLIAEVHAAFKRHVEREEAPVVV
MucR_Smeliloti -MTETSLGTSNELLVELTAEIVAAYVSNHVVPVAELPTLIADVHSALNNTTAPAPVVVPV
MucR_Sfredii -MTETTLGASNELLAELTAEIVAAYVSTHVVPGAELPTLIADVHSALNNATAPAPVIAPI
Ros_Atumefaciens -MTETAYGNAQDLLVELTADIVAAYVSNHVVPVTELPGLISDVHTALSGTSAPASVAVNV
Ml1_Mloti --MTEEADKNIDTLIELTADVVSAYVSNNPVPVGDLPALIGQVHAALKGTAG-FVSAAKP
Ml5_Mloti --MTEETESKADNLIELTAHVVSAYVSNNPVPVGELPGLIGQIHIALKGTAG-GAAPEKS
Ml3_Mloti --MKELSNIEDKTVIELTADIVSAYVGNNPLPASGLPDLIASVSASVRKLAG-AVVVESP
MucR2_Ccrescentus -------MEDQSDLIEMTAGIVSAYVGNNVVSTADLPALIKQVHAALANVGAPDAEAAPT
MucR1_Ccrescentus -------MEDKATLIELTAEIVANYVANNSTPVSELPALIRATHDALAGIGSPPAPTVEV
 : .:** :*: **..: ** ** :.
Ml2_Mloti -KQKPAVNPKRSVHDDYIVCLEDGKKFKSLKRHLMTHYDLTPDQYREKWNLDPSYPMVAP
MucR_Babortus EKPKPAVNPKKSVHDDYIVCLEDGKKFKSLKRHLVTHYNMTPEQYREKWDLDPNYPMVAP
MucR_Smeliloti EKPKPAVSVRKSVQDDQITCLECGGTFKSLKRHLMTHHNLSPEEYRDKWDLPADYPMVAP
MucR_Sfredii EKPKPAVSIRKSVQDDQITCLECGGAFKSLKRHLMTHHNLSPEDYREKWDLPADYPMVAP
Ros_Atumefaciens EKQKPAVSVRKSVQDDHIVCLECGGSFKSLKRHLTTHHSMTPEEYREKWDLPVDYPMVAP
Ml1_Mloti EALEPAVPIRKSVTPDYIICLDDGKKFKSLKRHLSTHHGLTPDEYRAKWHLPADYPMVAP
Ml5_Mloti EALKPAVPIRKSVTPDYIISLEDGKKFKSLKRHLATHYGLTPDEYRAKWELPADYPMVAP
Ml3_Mloti -SLVPAVNPKKSVFPDYIICLEDGKKFKSLKRHLRTDYGLSPDDYRAKWGLPPDYPMVAP
MucR2_Ccrescentus -PKEPAVPVKKSITPDYLICLEDGRKFKSLKRHLRTKYDMTPEDYRAKWGLPKDYPMVAP
MucR1_Ccrescentus VTKATPAQIRKSITPEALISFEDGKPYKTLKRHLTTHG-MTVAEYKAKWGLPNDYPTTAP
 ... ::*: : : .:: * :*:***** *. :: :*: ** * .** .**
Ml2_Mloti NYAAARSQLAKKMGLGRKRKAR---------------
MucR_Babortus NYAAARSRLAKKMGLGRKPKDA---------------
MucR_Smeliloti AYAEARSRLAKEMGLGQRRKRRGK-------------
MucR_Sfredii AYAEARSRLAKEIGLGERRKRRGK-------------
Ros_Atumefaciens AYAEARSRLAKEMGLGQRRKANR--------------
Ml1_Mloti NYAAARSALAKTMGLGRKPKEPEARTRKKAAA-----
Ml5_Mloti NYAAARSALAKTMGLGRKPKEPETPAPAKRARKKAAA
Ml3_Mloti NYSATRSALAKSTGLGRKPAAAPAAVAKKGKAKA---
MucR2_Ccrescentus NYAEARSNLAKQMGLGQGGRKPARKAK----------
MucR1_Ccrescentus AYSEARSQMAKALGLGQGGRKGKTTRGRKG-------
 *: :** :** ***.

## Slide 8
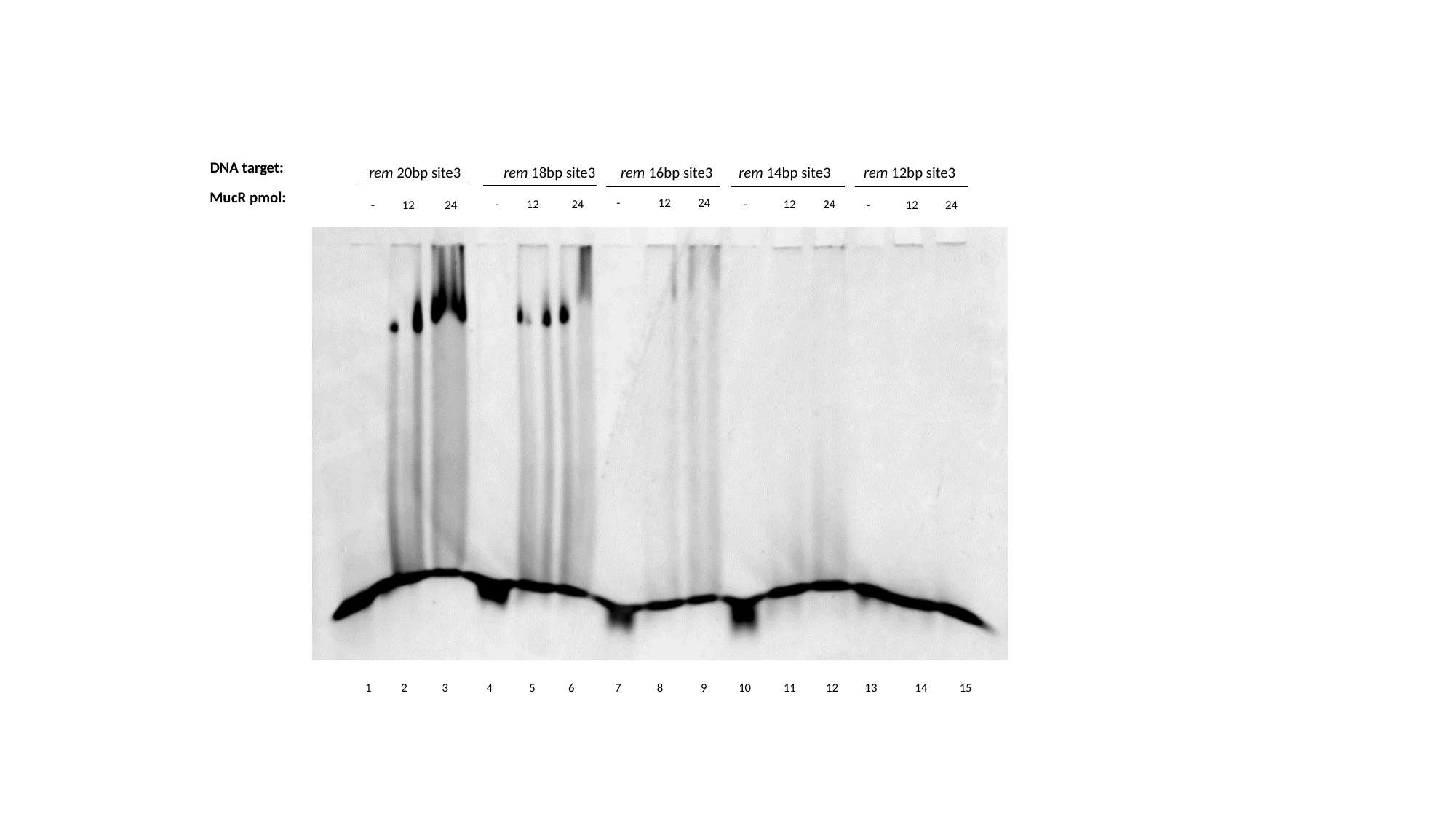

DNA target:
rem 20bp site3
rem 18bp site3
rem 16bp site3
rem 14bp site3
rem 12bp site3
MucR pmol:
- 12 24
- 12 24
 - 12 24
 - 12 24
- 12 24
1
2
3
4
5
6
7
8
9
10
11
12
13
14
15
